# Keeper-Animal Interactions: Differences between the Behaviour of Zoo Animals Affect Stockmanship

**DOI:** 10.1371/journal.pone.0140237

**Published:** 2015-10-28

**Authors:** Samantha J. Ward, Vicky Melfi

**Affiliations:** Paignton Zoo Environmental Park, Devon, United Kingdom; Harvard University Faculty of Arts and Sciences, UNITED STATES

## Abstract

Stockmanship is a term used to describe the management of animals with a good stockperson someone who does this in a in a safe, effective, and low-stress manner for both the stock-keeper and animals involved. Although impacts of unfamiliar zoo visitors on animal behaviour have been extensively studied, the impact of stockmanship i.e familiar zoo keepers is a new area of research; which could reveal significant ramifications for zoo animal behaviour and welfare. It is likely that different relationships are formed dependant on the unique keeper-animal dyad (human-animal interaction, HAI). The aims of this study were to (1) investigate if unique keeper-animal dyads were formed in zoos, (2) determine whether keepers differed in their interactions towards animals regarding their attitude, animal knowledge and experience and (3) explore what factors affect keeper-animal dyads and ultimately influence animal behaviour and welfare. Eight black rhinoceros *(Diceros bicornis)*, eleven Chapman’s zebra *(Equus burchellii)*, and twelve Sulawesi crested black macaques *(Macaca nigra)* were studied in 6 zoos across the UK and USA. Subtle cues and commands directed by keepers towards animals were identified. The animals latency to respond and the respective behavioural response (cue-response) was recorded per keeper-animal dyad (n = 93). A questionnaire was constructed following a five-point Likert Scale design to record keeper demographic information and assess the job satisfaction of keepers, their attitude towards the animals and their perceived relationship with them. There was a significant difference in the animals’ latency to appropriately respond after cues and commands from different keepers, indicating unique keeper-animal dyads were formed. Stockmanship style was also different between keepers; two main components contributed equally towards this: “attitude towards the animals” and “knowledge and experience of the animals”. In this novel study, data demonstrated unique dyads were formed between keepers and zoo animals, which influenced animal behaviour.

## Introduction

“Stockmanship” is a term used by many to describe the management of animals with a good stockperson someone who does this in a in a safe, effective, and low-stress manner for both the stock-keeper and animals involved. It has become a focus of discussions due to the significant impact it has on animal husbandry, productivity and welfare standards for livestock, with poor stockmanship leading to lower productivity and animal welfare [1]. Strategies have been proposed to appropriately recruit and train stock-keepers to ensure minimum standards of animal care are reached by maintaining good stockmanship levels as it has been seen to be linked to their personality, skills and experience and these are areas that can be empirically tested [2]. Investigations into stockmanship began within the agricultural industry and revealed that negative human-animal interactions (HAI) led to a reduction in productivity, reproduction rates and increased an animal’s fear of humans in dairy cows and pigs for example [3, 4, 5]. Studies indicated that negative handling (including slapping, pushing, shouting or scare tactics to move the animals on) of pigs, reduced growth, feed conversion efficiency and pregnancy rates [6, 7] and increased basal free cortisol rates and size of adrenal glands; all of which are indicative of a stress response [7, 8]. Pedersen *et al*. [9] also found that increased fear of humans in oestrous sows reduced their attraction to boars when in the presence of humans, thereby reducing the sow’s potential breeding capacity and production rates. Hemsworth [4] showed that stockpeople with a negative attitude towards animals were partly responsible for declines in animal productivity.

In contrast to the negative ramifications for animal welfare of negative HAI, positive HAI have been associated with increased productivity in pigs [3], cows [10–12] and other livestock studied [3]. The impact of positive HAI in broiler chickens, which was provided in the form of one minute daily contact from the handlers for 14 days prior to experiments taking place, was observed to result in greater growth rates, feed conversion efficiency and antibody response measured in the blood compared to broiler birds that received lower levels of human contact [13].

The study of stockmanship has primarily focused on the implications of HAI with domestic livestock; however recent studies have started to investigate the impact of HAI on the behaviour and welfare of captive wildlife. For example, when provided with an increased level of positive HAI from laboratory care staff, chimpanzees *(Pan troglodytes)* performed more allo-grooming, which was considered to indicate good animal welfare. Furthermore, the chimpanzees were also observed to perform less behaviour indicative of stress, such as abnormal oral behaviours like regurgitation and re-ingestion, inactivity, and excessive reactivity to the social displays of neighbouring chimpanzees [14]. Manciocco *et al*., [15] found that positive human interactions directed towards common marmosets *(Callithrix jacchus)*, were associated with an increased level of grooming and playful activities, and lower level of self-scratching which together were considered indicative of increased welfare. Melfi and Thomas [16] showed that positive HAI provided by zoo keepers to a group of captive Abyssinian colobus monkeys *(Guereza kikuyuensis)*, through the provision of positive reinforcement training, reduced the frequency of the colobus’ initiated interactions with both familiar (keepers) and unfamiliar (other staff and visitors) people, but did not affect the animal’s activity budgets or social interactions between individuals; both considered beneficial for zoo animal welfare where it is hoped the animals will live autonomously of people. Ward and Melfi [17] also found that the inclusion of positive reinforcement training into the general husbandry regime for several animals, provided the opportunity to increase the number of positive interactions initiated by keepers to animals; they suggested that this elevated level of positive HAI may reduce the perceived fear towards humans and positively contribute to the creation of positive human-animal relationships (HAR).

Hosey [18] developed a model to explain how HAR might develop in a zoo setting, where he distinguished between familiar (zoo professionals) and unfamiliar (zoo visitors) humans. Like the HAR model for agricultural animals [19], it was suggested that the development of a HAR was determined by the level of fear towards humans. The form and frequency of HAI initiated towards zoo animals would determine the type of relationship which resulted; negative, neutral or positive HAI would be expected to lead to negative, neutral or positive HAR. Several studies have collected some data to test this model for unfamiliar humans (zoo visitors) but to date, few studies have explored the impact of familiar humans in this context (see review papers [20, 21]).

A good stockperson (defined as managing livestock in a safe, effective, and low-stress manner for both the stock-keeper and animals involved) is expected to initiate positive interactions towards the animals; but are there factors which determine whether someone is likely to be a good stockperson? Hemsworth *et al*., [2] and Boivin *et al*., [3] found that stockpeople displayed positive HAI when they had a positive attitude towards the animals they worked with. This was evidenced by them describing the animals in a positive manner when questioned about them and directing communication (vocal or other) to them in a positive tone or manner. As attitudes are based on cognitive, affective and behavioural information, they can change in different circumstances [22]; thus poor stockpeople given the appropriate training can become good stockpeople [19]. Hemsworth and Coleman [1] later suggested that other factors could also predict the quality and style of a stockperson, including; competency within the work place, motivation, attitude towards work and certain personality traits are job-related fundamentals for good stockmanship.

Stockmanship in zoos is an important area of research, as it provides data on which to base HAI and ensure high animal welfare standards. Phillips and Peck [23] examined the role of personality in keeper-tiger interactions and found that when keepers were more angry and neurotic they were less likely to perform positive interactions towards captive tigers under their care. They suggested that self-rated keeper personality impacted on the keeper-tiger interaction more than the tigers’ personality, and that it likely influenced stockmanship style. Carlstead [24] described how certain stockmanship styles might induce fear in zoo animals. She measured the animals’ response rate to certain cues and commands provided by the keepers, after which the animals were expected to perform a specified behaviour. Results suggested that the animals studied (maned wolf, *Chrysocyon brachyurus* and cheetah, *Acinonyx jubatus*) showed increased aggression or apprehension when keepers made unexpected noises or movements. It was concluded that stockmanship style affected zoo animal behaviour, but more information was needed to investigate factors which might influence it, for example, keeper attitudes and behaviours towards the animals in their care, and how these might affect zoo animal welfare [24].

Studies cited above suggest that HAI between keepers and zoo animals differ between keeper-animal dyads as a consequence of different measurable components that previously have been linked to stockmanship such as attitude and personality and thus as a consequence unique keeper-animal dyads may be formed. Previous findings support that there is likely to be variation between individual animal responses to cues and commands provided by keepers, in addition to variation in responsiveness between species [17, 24]. High latencies in response to certain cues and commands provided by keepers could indicate that animals have a high level of fearfulness towards humans. Animals that have developed a more positive HAR with certain keepers, through an increased number of positive HAI, are less likely to show fear towards their keepers and therefore perform the required behaviours more readily. Following the idea that understanding the relationship between keepers and animals (i.e. style of handling and their dyads) could benefit the welfare of animals, the aims of this study were to: (1) investigate if unique keeper-animal dyads are formed in zoos, (2) determine whether keepers differed in their interactions towards animals and (3) explore what factors of stockmanship affect keeper-animal dyads and ultimately the behaviour and welfare of the animals.

## Methods

The study was successfully approved with ethical clearance from the Whitley Wildlife Conservation Trust (WWCT) ethical committee and followed the ARRIVE guidelines [25] and the British Psychological Society Code of Human Research Ethics [26] where necessary. This study followed a single-blind experimental method, thus keepers were initially and during data collection unaware of the true nature of the study as this may have impacted on their responses to the questionnaire and behaviour towards the animals (Hawthorne effect [27]). During a verbal de-briefing at the end of the study, keepers were informed about the true goal of the study, and given the opportunity to ‘opt out of the study’ or to ‘give permission for their data to be used in the study’; all keepers gave verbal permission for their data to be used and were positive towards the goal of the study. Written consent was not obtained as it was not a requirement for the WWCT ethics committee or thought necessary by the participating organisations; indeed only positive feedback was provided both by the study subjects and the participating organisations. Had any participants wished to not be part of the study, data recorded of their interactions with the animals and other data pertaining to them would have been destroyed; in accordance with the WWCT ethics committee.

Research managers at all anonymous institutions were fully aware of the study’s aims and objectives and gave permission prior to data collection. Once data had been collected, permission from each location was granted before data was included within the manuscript. Institutions where study subjects were observed and data collected have been kept anonymous, to further protect the identity of the keepers involved.

### Subjects, housing and husbandry

Eight black rhinoceros *(Diceros bicornis)* aged between 6 and 10 years old, eleven Chapman’s zebra *(Equus burchellii)*, aged between 4 and 8 years old; and twelve Sulawesi crested black macaques *(Macaca nigra)* aged between 3 and 12 years old were studied; only adults were included in data collection. The animals were maintained in five British and one American zoo (Table 1).

**Table 1:** Division of species at the different institutions showing numbers of males and females (♂.♀) and the number of keeper-animal dyads recorded (number of keepers x number of animals recorded).

	*Diceros bicornis*		*Equus burchellii*		*Macaca nigra*	
	♂.♀	dyads	♂.♀	dyads	♂.♀	dyads
**UK 1**	1.1	6	-	-	2.2	12
**UK 2**	2.2	12	-	-	-	-
**UK 3**	-	-	2.2	12	2.2	12
**UK 4**	-	-	2.3	15	-	-
**UK 5**	-	-	2.0	8	2.2	12
**USA 1**	1.1	4	-	-	-	-

Study animals were selected to include different mammal species that were managed in accordance to the training conditions required by Ward and Melfi [17], and held in the same institutions. All enclosures within each institution were cleaned daily in the morning before the zoo opened to the public. This procedure involved cleaning the outdoor enclosure whilst the animals were locked indoors, then the animals were moved to their outdoor enclosure and the vacated indoor enclosure was cleaned; the time access was provided between the indoor and outdoor enclosure areas varied between zoos.

### Data Collection

#### Behavioural data

During preliminary observations of a keeper’s working day (approx. eight hours), conducted for each species, most HAI were consistently observed during the first 2 h (08.00–10.00 am), and last hour (16.00–17.00 pm) of the day; this corresponded with times when animals were being moved between their indoor/outdoor enclosures. Consequentially these 3hrs were used to observe all keeper cues and commands (CC) visible to the observer (visual, auditory, contextual), directed towards the study animals, and the animals’ respective behaviours were recorded. Three CC common to all species and zoos, which were not part of a training programme as previously described, were identified which were (i) a nonverbal CC, where the keeper approached the enclosure without calling the animals' names, but the animals were expected to respond to the opening of keeper-controlled doors; (ii) a verbal CC, where the keeper ‘asked’ the animal to move to the outside enclosure from the inside enclosure; (iii) and the final verbal CC, where the keeper ‘asked’ the animal to move to the inside enclosure from the outside enclosure [17]. The latency between the keeper performing the CC and the animals’ behavioural response was measured, as well as any escalation to the CC; the extent to which CC were repeated and how, i.e. in a negative or positive tone or associated with actions; the latter was subjectively rated on a cumulative scale resulting in an escalation score. The socially grouped animals were individually identified, and observations recorded per individual. Latencies for each animal to perform the required behaviour following all cues/commands were recorded per keeper–animal dyad (Table 1). Sufficient observations were undertaken to ensure that each CC was observed 8 times for each keeper–animal dyad (n = 93, total number of keepers 27; Table 1) over a total period of four months.

#### Stockmanship

Questionnaires (S1 Text), similar to those constructed by Hemsworth [4], were designed to assess the measurable aspects of stockmanship including attitude, animal knowledge and experience. Thirty questions investigated three main areas including job satisfaction, attitude towards the animals in the care of keepers, and how they perceived their relationship with the animals. Questionnaires followed a five-point Likert Scale design. Demographic information was also sought, focused on the level of education, length of experience working with the species and details of other animal-related work experience.

### Data Analysis

All data were coded to ensure the identity of keepers, zoos and animals remained anonymous and were not identified in data analyses or interpretation. IBM SPSS Statistics, Version 22 was used for all statistical analysis.

Data were separated at the CC and species level. Behavioural data were not normally distributed so a log transformation was applied to the observed latencies, thus a General Linear Model could be performed in the form of three Two-way ANOVAs one per species. These were used to identify differences in the animals’ latencies to respond, where animal and keeper were fixed factors in three two way ANOVAs one for each species.

An exploratory Factor Analysis (EFA) was employed to reduce the original 30 questionnaire statements with an oblique rotation (varimax) as the questions were linked and strongly related. A paired t-test was then used to identify any differences between the calculated component scores.

## Results

### Latencies

There was a significant difference in the animals’ latency to respond (S1 Table) with the appropriate behaviour after the provision of all 3 CC, in all three species (Table 2). There was also a significant interaction between keeper and animal, in the rhinos latency to respond appropriately after all three CC (C1: F_6,72_ = 19.074, p<0.001; C2: F_6,72_ = 14.555, p<0.001; C3: F_6,72_ = 15.845, p<0.001).

**Table 2:** Results from a two-way ANOVA showing main effects and interaction of the two main effects to show the variation in the performance of the appropriate behaviours of the macaques, zebra and rhino according to the different cues.

Species	Cue	Keeper		Animal		Keeper*Animal	
		F value	sig	F value	sig	F value	sig
**Macaque**	1	9.995	.000*	1.475	.164	.240	.999
	2	13.788	.000*	4.023	.000*	.848	.624
	3	2.381	.040*	3.253	.001*	.533	.921
**Zebra**	1	27.923	.000*	1.156	.328	.391	.980
	2	29.242	.000*	1.386	.206	.264	.998
	3	3.912	.002*	2.773	.006*	.569	.897
**Rhino**	1	25.737	.000*	31.236	.000*	19.074	.000*
	2	14.477	.000*	5.716	.000*	14.555	.000*
	3	13.016	.000*	9.544	.000*	15.845	.000*

* highlights statistical significance.

### Stockmanship

The EFA revealed there was a high degree of multicolinerarity in the responses to these statements so responses to 18 questions which scored >0.9 or <0.3 were therefore removed from analysis [28]. The remaining 12 statements (Table 3) were used to create components describing stockmanship based on the criterion of having an eigenvalue greater than 1.00. The Kaiser–Meyer–Olkin (KMO) measure verified the sampling adequacy for the analysis with KMO = 0.727 which was well above the acceptable limit of 0.5 [29]. The KMO value and the Bartlett’s test of sphericity (χ^2^_35_ = 193.37, p < 0.001) indicated that the correlations between items were sufficiently large enough for an EFA to be performed.

**Table 3:** Summary of the exploratory factor analysis results from all 27 keeper questionnaires. Showing the Pattern Matrix with principal axis factoring extraction method and an oblimin rotation with Kaiser Normalisation. The Eigenvalue, percentage of variance and Cronbach’s alpha score are also provided for the two components.

Questions	Rotated Factor Loadings	
	Negative Attitude	Knowledge & Experience
I am not generally patient with them	.856	
They are stubborn	.827	
they are not pleasant to work with	.817	
They are not friendly	.761	
People make too much fuss over animals feelings	.756	
They are bad tempered	.734	
They are not clever	.648	
They are easy to manage		.915
I have a lot of experience with them		.824
I would like to learn more about management of them		-.687
I feel I still have a lot to learn about them		-.576
I don’t know much about disease in them		-.364
Eigenvalue	6.183	2.011
Percentage of Variance	62.43	17.11
α	.83	.71

Keepers’ responses were significantly different to all 12 questionnaire statements monitoring stockmanship style: “I am generally patient with them [the animals]” (t_22_ = 27.177, p<0.001), “They [the animals] are clever” (t_22_ = 21.828, p<0.001), “They [the animals] are friendly” (t_22_ = 17.702, p<0.001), “I don’t know about diseases of them [the animals]” (t_22_ = 17.179, p<0.001), “They [the animals] are pleasant to work with” (t_22_ = 16.539, p<0.001), “They [the animals] are bad tempered” (t_22_ = 14.469, p<0.001), “I have lots of experience with them [the animals]” (t_22_ = 12.697, p<0.001), “I still have a lot to learn” (t_22_ = 11.013, p<0.001), “They [the animals] are stubborn” (t_22_ = 9.919, p<0.001), “I would like to learn more about management of them [the animals]” (t_22_ = 9.721, p<0.001), “People make too much fuss over animal’s feelings” (t_22_ = 9.650, p<0.001), “They [the animals] are not easy to manage” (t_22_ = 8.429, p<0.001).

From the questionnaire data, where 12 from the original 30 statements were used, EFA identified two main components which contributed towards stockmanship style, explaining 79.54% of the total variance in the questionnaire responses (Table 3). The components were “attitude towards the animals” and “knowledge and experience” of the animals which keepers worked with. Table 3 includes the pattern matrix used for the production of these two components. When the scores were investigated further, both of these components were found to equally influence the keepers’ stockmanship (t_21_ = 0.940, p>0.05).

## Discussion

The animals’ latency to respond to CC provided by the keepers showed that all three study species reacted significantly differently to different keepers. This suggests animals were behaving differently in response to different keepers; i.e. some keepers received quicker responses than others. This variation in the animals’ reaction to keepers supports the findings of Carlstead [24] and Ward and Melfi [17]. They found that the HAR between keepers and animals differed depending on the HAI experienced. The current study was able to demonstrate that individuals of all study species reacted differently to different CC provided by keepers however with the rhinos there were consistent interactions suggesting that their keeper-animal relationships were unique. Perhaps some keepers were themselves inconsistent in their interactions with the study species, hence the animals reacted differently to CC but not necessarily to keepers. However rhino keepers might have been more consistent in their provision of CC, hence the rhinos were able to predict and respond differently to different keepers. Ward and Melfi [17] indicated that socially housed species responded significantly quicker to keeper CC in comparison to solitary species. It could therefore be possible that solitary housed species are more influenced by HAI and are more likely to form HAR due to their solitary lifestyle. This could be linked to the animals’ behavioural ecology as solitary species may not respond to CC as readily a social species.

The self-reporting questionnaires were measuring how keepers perceived their own attitudes, knowledge and experience as this has been seen to correlate with animal directed behaviours in previous research [1–3, 17]. Results indicated that there were significant differences in the responses provided by the keepers, suggesting that their attitudes to the animals in their care, their perception of their relationship with them, and their knowledge and work experience were different; all of which are components which likely to contribute to stockmanship. These results echo similar findings from research in the agricultural industry [1–3] and a study in zoos [17] where there were several factors which were seen to influence stockmanship with domestic and some exotic species. The positive or negative extent to which any one of these components was expressed by any keeper is where they lay along the spectrum for that particular component.

Factors contributing to different stockmanship styles were good indicators of the differences found in the responses of the animals to the keepers CC. Research on stockmanship style or studies analysing the factors contributing to different styles have not been conducted using zoo keepers as a study population. Results from the current study suggested there were two components which can be used to describe keeper stockmanship including the attitude towards the animals and knowledge and experience of the animals under their care. According to previous research a good stockperson shows low levels of escalation and has animals which respond to their CC faster; therefore escalation was less likely in keepers who had a positive attitude towards the animals in their care. Good stockmanship ability was likely if the keepers had a positive attitude toward the animals they worked with and they had extensive experience and species knowledge. With the differences between the keepers, the animals responded differently. With both agricultural and zoo species, differences in the human characteristics (escalation, attitudes, knowledge, and experience) may contribute to differences in the interaction quality as well as the frequencies. Therefore the differences recorded between the animals responses to humans, demonstrates that the animal has also had a big impact on how that interaction operates.

In the current study keepers’ age ranged from 22 to 45 years old, and they had varied amount of work-related experience with the study species, from 8 months up to 23 years. The keepers also varied in the formal education they had received and whether this related to animals; younger keepers had qualifications ranked in the current study at level six (UK based Bachelor’s degree) and seven (UK based Master’s degree), compared to older keepers who had level two (e.g. UK based National Diploma) and level three qualifications (e.g. UK based Extended Diploma), or had only recently completed their studies. It is for this reason that separation of experience and knowledge is not possible as knowledge is not necessarily knowledge gained from a specific course but could be on the job training or knowledge gained through years of experience.

This study demonstrates that positive attitudes towards the animals, good subject knowledge and familiarity with the species are linked to positive behavioural responses in a range of exotic species. Staff with negative attitudes towards animals or those which lack a good level of experience or education should be avoided in keepers working with exotic species. This will ensure a positive relationship between the animal and the keeper which in turn reduces stress and increases animal welfare.

## Conclusion

Of the many factors that can affect zoo animal behaviour, particular attention should be paid to human-animal interactions that occur on a daily basis between keepers and animals as they are a vital and permanent feature of the animals’ lives. With this in mind this study was able to demonstrate that keeper-animal dyads were formed and keepers were found to have different stockmanship styles, which were influenced by keeper attitude and knowledge of the animals in their care, and their work experience.

## Supporting Information

S1 Text Stockmanship Questionnaire. Questionnaire completed by the keepers working with the animals being observed. The example attached is written for the Sulawesi black crested macaque *(Macaca nigra)* keepers, with the others being exactly the same but with the species changed on the questionnaire.
(DOC)

S1 Table Latency data for animals and keepers. Where MK = macaque keeper, RK = rhino keeper, ZK = zebra keeper, M = macaque (individual), R = rhino (individual), Z = zebra (individual).
(XLSX)
