# Supplementary material for: Keeper-Animal Interactions: Differences between the Behaviour of Zoo Animals Affect Stockmanship
Source: PLoS One. 2015 Oct 28;10(10):e0140237. doi: 10.1371/journal.pone.0140237 (PMC4624973; doi:10.1371/journal.pone.0140237)
Supplement: S1 Text — Questionnaire completed by the keepers working with the animals being observed. The example attached is written for the Sulawesi black crested macaque (Macaca nigra) keepers, with the others being exactly the same but with the species changed on the questionnaire. (DOC) [file pone.0140237.s001.doc]

**Personal Information**

Name_____________________________________

Position___________________________________

Age_____ Sex: M F

Months/ years experience working with Sulawesi macaques?____________________________

with these macaques?____________________________

as a keeper?____________________________

How many days per week are you responsible for the care of the SM’s?___________________

How many hours per day do you spend around the SM’s?_______________________________

How many keepers work with the SM’s?__________________­­­­­__________________________

Do you hold any relevant qualifications? If so please list________________________________

_____________________________________________________________________________

**Using a five point scale, where 1 = disagree strongly to 5 = agree strongly and N/A means not applicable, please circle the answer that most closely matches your personal opinion:**

1. SM’s are stubborn animals 1 2 3 4 5 N/A
2. SM’s are not friendly animals 1 2 3 4 5 N/A
3. SM’s respond to being talked to 1 2 3 4 5 N/A
4. I like working with SM’s 1 2 3 4 5 N/A
5. I don’t know much about disease in SM’s 1 2 3 4 5 N/A
6. I get along with my colleagues 1 2 3 4 5 N/A
7. SM’s are bad tempered animals 1 2 3 4 5 N/A
8. SM’s are pleasant to work with 1 2 3 4 5 N/A
9. SM’s don’t respond to being stroked 1 2 3 4 5 N/A
10. SM’s are smelly 1 2 3 4 5 N/A
11. I chose to work with SM’s 1 2 3 4 5 N/A
12. People often make too much fuss over the feelings of animals 1 2 3 4 5 N/A
13. I am generally patient with SM’s 1 2 3 4 5 N/A
14. SM’s are clever animals 1 2 3 4 5 N/A
15. Working as a zoo keeper has met my expectations 1 2 3 4 5 N/A
16. SM’s are dangerous animals to work with 1 2 3 4 5 N/A
17. SM’s are ugly 1 2 3 4 5 N/A
18. I look forward to lunch and tea breaks 1 2 3 4 5 N/A
19. Little experience is required to work with SM’s 1 2 3 4 5 N/A
20. SM’s don’t feel pain 1 2 3 4 5 N/A
21. SM’s are greedy 1 2 3 4 5 N/A
22. SM’s are interesting animals 1 2 3 4 5 N/A
23. SM’s are docile animals 1 2 3 4 5 N/A
24. SM’s are not easily frightened 1 2 3 4 5 N/A
25. I have a lot of experience with SM’s 1 2 3 4 5 N/A
26. I feel that I still have a lot to learn about SM’s 1 2 3 4 5 N/A
27. SM’s are not easy to manage 1 2 3 4 5 N/A
28. I feel that I am sensitive to the mood of the SM’s 1 2 3 4 5 N/A
29. I do not enjoy spending time with the SM’s 1 2 3 4 5 N/A
30. I would like to learn more about the management of SM’s 1 2 3 4 5 N/A
